# Efficacy and safety of massage for postoperative stress in colorectal cancer patients: a randomized, controlled, three-arm trial

**DOI:** 10.3389/fonc.2025.1439420

**Published:** 2025-02-05

**Authors:** Paul G. Werthmann, Dirk Cysarz, Melanie Jungbluth, Ann-Kathrin Lederer, Gergana Nenova, Roman Huber, Monique van Dijk, Gunver S. Kienle

**Affiliations:** ^1^ Center for Complementary Medicine, Department of Medicine II, Medical Center—University of Freiburg, Faculty of Medicine, University of Freiburg, Freiburg, Germany; ^2^ Institute for Applied Epistemology and Medical Methodology, University of Witten/Herdecke, Freiburg, Germany; ^3^ Research Group Integrative Medicine, Department of General and Visceral Surgery, University Hospital Ulm, Ulm, Germany; ^4^ Integrated Curriculum for Anthroposophic Medicine, Faculty of Health, Witten/Herdecke University, Witten, Germany; ^5^ Department of General, Visceral and Transplant Surgery, University Medical Center of the Johannes Gutenberg University, Mainz, Germany; ^6^ Department of General and Visceral Surgery, Center of Surgery, Medical Center - University of Freiburg, Freiburg, Germany; ^7^ Department of Internal Medicine, Section Nursing Science, Erasmus University Medical Center, Rotterdam, Netherlands

**Keywords:** colorectal cancer, rhythmical embrocations, massage, stress, heart-rate-variability

## Abstract

**Purpose:**

The purpose of this study was to investigate the effect of rhythmic embrocation (RE), a massage technique, on postoperative stress levels (measured by heart rate variability) in colorectal cancer surgery patients compared to empathic conversation.

**Methods:**

The study included 68 patients who were randomized into three groups: one received RE from professionals, another from students, and the third received empathic conversations. Stress was quantified using heart rate variability before and after the interventions.

**Results:**

The standard deviation of the heartbeat intervals (SDNN) increased more in the professional RE group 9.12 ms (IQR 3.59-12.3 ms) than in the other groups: student RE group 5.68 ms (-0.66-7.5 ms), empathic conversation group 6.64 ms (-1.49-7.38 ms); hence stress decreased more in the professional RE group, although not statistically significant (p= 0.21). Other factors like sleep quality, nausea, pain, and mood did not differ significantly between the groups. No complications were associated with the interventions.

**Conclusion:**

RE was safe and a statistically significant superiority of RE on postoperative stress compared to empathic conversations could not be found. Due to high inter- and intraindividual variability a clear pattern of response of the secondary outcomes to RE in comparison to empathic conversations could not be found. The study was limited by a small sample size, high patient variability, effective co-interventions for sleep, pain and nausea, and by an imbalance between groups. The study indicates that future research on RE should focus on a more narrowly defined patient population, increase the sample size, and select comparison groups that are clearly distinct from each other as well as a clinical context with fewer confounding factors. Furthermore, the patient’s preferences and previous experiences with massage therapy should be considered.

**Clinical trial registration:**

German Clinical Trials Register (www.drks.de), identifier DRKS00023407.

## 1 Introduction

Colorectal cancer is the fourth most commonly diagnosed cancer, after breast, lung and prostate cancer. About 80% of colorectal cancers are diagnosed at a localized stage, when surgery can be curative (1).

Stress responses to surgery can be assessed by questionnaires, by monitoring sympathetic nervous system activation, pituitary hormone secretion, insulin resistance, immunologic and hematologic changes, and heart rate variability (HRV) (2, 3). Standards for HRV measurement have been developed (4), and HRV is increasingly being used in stress research (3, 5–17). Stress can have a detrimental effect on surgical recovery, highlighting the importance of identifying methods to alleviate it (18).

The implementation of stress-reducing techniques could be beneficial. One such technique is rhythmical embrocation (RE), a manual massage therapy, which was developed in the early 20^th^ century by the physicians Ita Wegman and Margarethe Hauschka (19). RE therapy involves the application of oils or emulsions that a nurse gently massages over the patient’s body in circular or linear strokes (19, 20). This type of massage may support relaxation, and was followed by reductions in heart rate, blood pressure, pain, and improvements in mood, particularly in patients with chronic pain (21, 22).

The primary objective of this study was to investigate the effect of RE-therapy compared to empathic conversation on stress levels in colorectal cancer patients after surgery, as quantified by parameters of HRV. Secondary objectives included investigating the influence of RE-therapy on sleep quality, nausea and vomiting, pain level, analgesics use, sleep medication, mood, degree of mobilization, and any adverse events related to the intervention.

## 2 Methods

### 2.1 Trial design

The study was conducted as a two-center, randomized, controlled, open-label, three-arm, parallel-group trial. Patients were randomized 1:1:1 to the three groups. The trial was originally designed as a single-center study. As recruitment slowed down during the COVID-19 pandemic, an additional center was added and randomization was performed using a block randomization method stratified by center.

### 2.2 Participants

Individuals diagnosed with colorectal cancer and scheduled for surgery were eligible for this study. Inclusion criteria: patients in an inpatient setting after colorectal cancer surgery; the ability to complete questionnaires or answer questions; age 18 years or older; legal competence; comprehension of the trial’s nature and related procedures, along with the capacity to comply with them; proficiency in the German language; provision of written informed consent, obtained in accordance with international guidelines and local laws. Individuals were excluded from the study if they exhibited any of the following conditions: severe surgical complications; severe cardiac complications; sepsis; cardiac conditions that might interfere with HRV analysis, such as severe arrhythmias or the presence of a pacemaker; severe psychiatric disorders; pregnancy or breastfeeding; clinically significant comorbidities that could critically affect the patient’s adherence to the protocol; hypersensitivity to skincare products; skin disorders in the back or feet area; isolated nursing; participation in a clinical trial within the three months preceding the start of this study, or simultaneous participation in studies interfering with the current study.

### 2.3 Settings and locations

The study was conducted in the surgical departments of the University Medical Center Freiburg and the Evangelisches Diakoniekrankenhaus Freiburg, both located in southwestern Germany.

### 2.4 Interventions

RE-therapy was administered in two of the 3 study groups – in one group by RE-professionals, in the other group by nursing students. The third group of patients received empathic conversations with a nursing student. The nursing students who administered RE had received training in three teaching units of 3.5 hours each from two certified professionals (23). RE was applied as recommended (24) and with standardized procedure with the team (for a detailed description see the *Description of the intervention* in the **Supplementary Material**).

The interventions were started on the evening of the day the patient was discharged from the intensive care unit (ICU) following surgery. Consequently, the interval between surgery and the commencement of the intervention varied in days according to the duration of the patient’s stay on the ICU.

The participants received RE on the back (while sitting on the edge of the bed or lying on their side) and the feet (while lying on their back) with 2 mL of high-quality pharmacopoeial almond oil (25) for approximately 10 minutes, scheduled between 8 and 10 am and again between 6 and 10 pm. The intensity of the back massage ranged from 1 to 3 on a scale where 1 represented ‘gentlest massage possible’, and 10 = ‘very intense massage`, while the feet massage ranged from 1 to 5. RE of the back and feet were selected in discussion with several RE experts regarding the setting and condition of the patients and the goal of reducing stress in this setting.

The control intervention involved a 10-minute empathic conversation conducted during the same time periods. A list of suggested questions and topics was provided to the health professional students conducting the interviews. Following the intervention, all patients were instructed to rest for a period of 20 minutes. The staff and patients in the same room were informed about the intervention and resting period and were kindly requested not to interrupt during these periods. A sign was posted on the door indicating ‘study intervention in progress, please do not disturb until x:xx’.

We designed the study with the three groups described above to discriminate an effect between RE and empathic attention and to further evaluate whether a difference in outcomes can be seen between experts performing RE and students performing RE after only a short training in RE.

### 2.5 Outcomes

The primary outcome, ‘level of stress’, was assessed by analyzing HRV recorded overnight (starting between 6 and 10 pm and stopping between 8 and 10 am) on days 1 and 5 (days are counted from arrival at normal ward after surgery and intensive care unit) using a one-channel electrocardiogram (ECG) device, the Bittium Faros 180 (26). The devices were installed on the patient by the study personnel (students, RE experts). The data were analyzed in accordance with the 1996 guideline of the Task Force of the European Society of Cardiology and the North American Society of Pacing and Electrophysiology (4). The primary metric utilized was the standard deviation of the N-N intervals (SDNN), expressed in milliseconds (ms), which serves to quantify the variability of heartbeats and indicates vagal activity. A higher SDNN indicates greater vagal activity and a lower stress level in the organism.

The secondary outcomes were evaluated as follows:

Sleep quality was evaluated using the Richards-Campbell Sleep Questionnaire (RCSQ) (27) on days 2, 3 and 6 after arrival on the normal ward after surgery and intensive care unit admission. The questionnaire generates a total score, ranging from 0 (indicating the worst possible sleep) to 100 (indicating the best sleep).

The severity of nausea experienced within the previous 24 hours was quantified on days 1, 3, and 6 using a 10-cm visual analogue scale (VAS), with endpoints ranging from “no nausea” to “worst imaginable nausea” (28, 29).

The intensity of pain experienced over the previous 24 hours was quantified on days 1, 3, and 6 using a 10-cm visual analogue scale (VAS), with endpoints ranging from “no pain” to “strongest imaginable pain.”

Information regarding the consumption of analgesics, sedatives, and emetics was obtained from the patient’s medical chart. Analgesics were classified according to the World Health Organization (WHO) analgesic ladder, with group 1 comprising non-opioid analgesics, group 2 low-potent opioids, group 3 high-potent opioids, and group 4 invasive pain management (pain catheter) (30).

Mood was assessed on days 1, 3, and 6 using the *Mehrdimensionaler Befindlichkeitsfragebogen* (MDBF), a validated questionnaire that measures well-being in terms of pleasantness, wakefulness, and calmness (31). The results are presented on a scale from 24, which represents the lowest mood, to 120, which represents the highest mood.

The activity level of the subjects was evaluated at baseline and on day 6 using the *Evaluationsbogen Mobilität (EboMo)*, a simple scoring tool for assessing activity level, especially in settings such as nursing homes. The responses were recorded on a scale from 14, indicating severely limited mobility, to 56, representing unrestricted mobility (32). Patients were queried about complications associated with the intervention at each encounter with the study team.

### 2.6 Change to trial outcomes

The initial definition of the primary outcome was the final HRV measurement. However, a significant proportion of ECG recordings could not be analyzed with respect to HRV parameters due to the presence of numerous artefacts in the ECG. Furthermore, due to the higher variability observed in HRV measurements as expected within the patient sample, we deemed it more meaningful to consider the change between these two measurements. This provides insight into the change in stress during the trial period. In the following section, we provide both outcomes.

### 2.7 Sample size

The sample size calculation was based on the data of a previous trial investigating perioperative stress with HRV parameters as outcome measure. In that trial, significant changes in the standard deviation of the distribution of normal-to-normal intervals (SDNN) had been recorded at different perioperative time points (5). The preoperative SDNN was 116 milliseconds (ms); on the first postoperative day it was 65 ms, and on postoperative day seven it was 87 ms [approximate values as the actual values are not given in the article text, but only displayed as a bar chart; the bar chart was converted into numbers using WebPlotDigitizer (33)]. Based on these figures, we assumed the median SDNN in the conversation group to be 80 ms on postoperative day 5. In the intervention groups, we assumed a faster recovery of the SDNN; i.e., 110 ms on day 5 (therefore we set SD = 30 ms). In the previous trial, the standard deviation was around 30 (SD = 30 ms) for all measurements. Based on a t-distribution, a two-tailed α=0.05 and a power of 0.8, we calculated a sample size of 17 patients per group, and thus 51 in total. Assuming a dropout rate of 20%, we therefore planned to include at least 60 patients.

### 2.8 Interim analyses

No interim analysis was performed and there was no early termination of the study.

### 2.9 Randomization and sequence generation

The allocation sequence was generated by an independent researcher using the website Randomization.com (34).

No stratification was used; we used block randomization by center.

### 2.10 Allocation and implementation

The participants were enrolled by a physician during the preoperative visit. Upon the patient’s arrival at the normal ward after surgery, the inclusion and exclusion criteria were re-evaluated. Upon the inclusion of a new patient, the investigator and the trial assistant were duly informed. Subsequently, the investigator or the trial assistant transmitted the sequential number of the new patient to the independent researcher via electronic means. Upon receipt of this information, the independent researcher transmitted the intervention code from the allocation list in response.

### 2.11 Blinding

It was not feasible to conduct a blind study of study staff who were directly involved in the intervention. Study staff providing RE were instructed not to disclose to the patients whether they were being treated by RE experts or students. The patients receiving RE were informed that they would not receive information regarding the identity of their treating RE professionals. The data collection and analysis were conducted in a manner that ensured blinding to the treatment group assignments.

### 2.12 Data management

HRV data were extracted and analyzed using Matlab (35). Data documented by patients themselves – with possible assistance of the study staff and data obtained by study staff in interviews was collected on paper and managed using REDCap (Research Electronic Data Capture) tools hosted at the University Medical Center Freiburg (36). REDCap is a secure, web-based application designed to support data capture for research studies. The application provides: 1) an intuitive interface for validated data entry; 2) audit trails for tracking data manipulation and export procedures; 3) automated export procedures for seamless data downloads to common statistical packages; and 4) procedures for importing data from external sources. The data was entered into REDCap 10.0.28 into fields with prespecified data ranges and plausibility checks. The datasets from Matlab and REDCap were then merged by patient ID in R version 4.2.0 (37).

### 2.13 Statistical methods

Data analysis was performed using R version 4.2.0 (37) an intention-to-treat approach using the full analysis set. A p-value < 0.05 was considered statistically significant. For the primary outcome ‘level of stress’, the SDNN was calculated from HRV during sleep based on recordings from day 1 and day 5, and the difference between these two measurements was calculated. The testing sequence was as follows: the mean of the empathic conversation group was compared with the mean of the expert RE group using the T-test; if the null hypothesis (no difference between the groups) could be rejected, the empathic conversation group was compared to the student RE group; if the null hypothesis could also be rejected in this comparison, the expert RE group was compared with the student RE group. If the data did not fulfil the criteria for parametric testing, non-parametric tests were used. The same procedures were applied to the secondary outcomes. The per-protocol group was defined in the study protocol as having no missing data in the primary outcome (HRV of day 1 and day 5) and having participated in more than 5 of 8 treatment sessions.

## 3 Results

**Figure 1:**
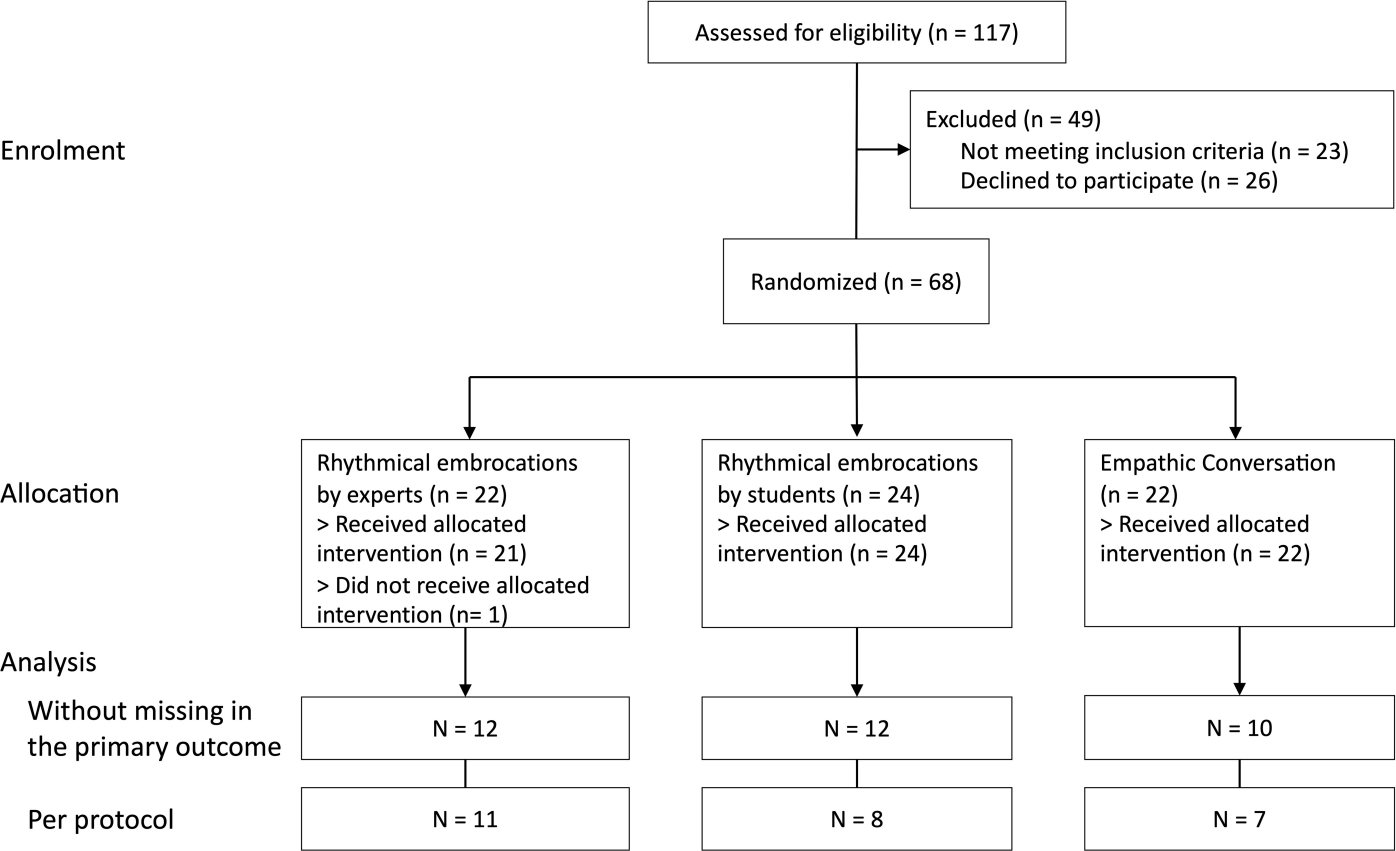
Patient flow diagram.

From January 2021 until January 2022, 68 patients were recruited and randomly assigned to one of the three study groups: 22 to the expert RE group; 24 to the student RE group and 22 patients to the empathic conversation group (**Figure 1**). The patients in the expert RE group received a median (range) of 6 (0-8) interventions, while those in the student RE group received 7 (2-8) interventions, and those in the empathic conversation group 7 (2-8) interventions. There were 5 drop-outs; 3 in the expert RE group and 1 in each of the other groups; reasons for dropping out in the expert RE group were complications of the surgical procedures (1 patient) and unspecified (2 patients); reasons for dropping out in the other groups were patients’ wishes (1 patient in each group). There were no intervention-specific drop-outs.

### 3.1 Baseline data

The demographic characteristics of the patients in each group are presented in **Table 1**. The mean age of the entire sample was 68 years (range 31-83 years), with an equal distribution of age across all three groups. The proportion of women in the entire sample was 39.7% (n=27). A significant difference in tumor localization was observed between the three groups (Kruskal Wallis: p = 0.02) especially between the student RE group and the empathic conversation group (Dunn’s-Test: p = 0.0076; see **Table 1**).

**Table 1:** Demographics of the patients, oncological and surgical characteristics per randomization group.

	Rhythmic embrocations by RE experts (n=22)	Rhythmic embrocations by students (n=24)	Empathic conversation (n=22)	Overall (N=68)
Sex				
female	12 (54.5%)	6 (25.0%)	9 (40.9%)	27 (39.7%)
Age (years)				
Mean (SD)	62.6 (12.7)	64.9 (13.1)	64.5 (9.40)	64.0 (11.7)
Marital Status				
single	5 (22.7%)	2 (8.3%)	1 (4.5%)	8 (11.8%)
married	14 (63.6%)	18 (75.0%)	13 (59.1%)	45 (66.2%)
widowed	2 (9.1%)	1 (4.2%)	2 (9.1%)	5 (7.4%)
divorced	0 (0%)	1 (4.2%)	1 (4.5%)	2 (2.9%)
Missing	1 (4.5%)	2 (8.3%)	5 (22.7%)	8 (11.8%)
Trial Center				
1 Univ. Medical Center Freiburg	14 (63.6%)	17 (70.8%)	15 (68.2%)	46 (67.6%)
2 Ev. Diakoniekh. Freiburg	8 (36.4%)	7 (29.2%)	7 (31.8%)	22 (32.4%)
Tumor Localisation				
Colon	13 (59.1%)	9 (37.5%)	17 (77.3%)	39 (57.4%)
Rectosigmoid	3 (13.6%)	5 (20.8%)	2 (9.1%)	10 (14.7%)
Rectum	6 (27.3%)	10 (41.7%)	3 (13.6%)	19 (27.9%)
Tumor Stage				
Stage 0-I	8 (36.4%)	8 (33.3%)	9 (40.9%)	25 (36.8%)
Stage II-III	6 (27.3%)	7 (29.2%)	5 (22.7%)	18 (26.5%)
Stage IV	8 (36.4%)	9 (37.5%)	8 (36.4%)	25 (36.8%)
Surgical Approach				
Laparoscopy	9 (40.9%)	12 (50.0%)	10 (45.5%)	31 (45.6%)
Laparatomy	13 (59.1%)	12 (50.0%)	12 (54.5%)	37 (54.4%)
Duration of surgical procedures (minutes)				
Median [IQR]	219 [128-288]	269 [156-403]	234 [113-284]	236 [121-314]
Blood loss (mL)				
Median [IQR]	50 [50-313]	200 [50-500]	75 [50-200]	100 [50-350]
Missing	4 (18.2%)	3 (12.5%)	2 (9.1%)	9 (13.2%)
Time on intensive care unit (days)				
Median [IQR]	2 [1-3]	1 [1-3]	1 [1-2]	1 [1-3]

### 3.2 Outcomes

#### 3.2.1 Primary outcome

**Figure 2:**
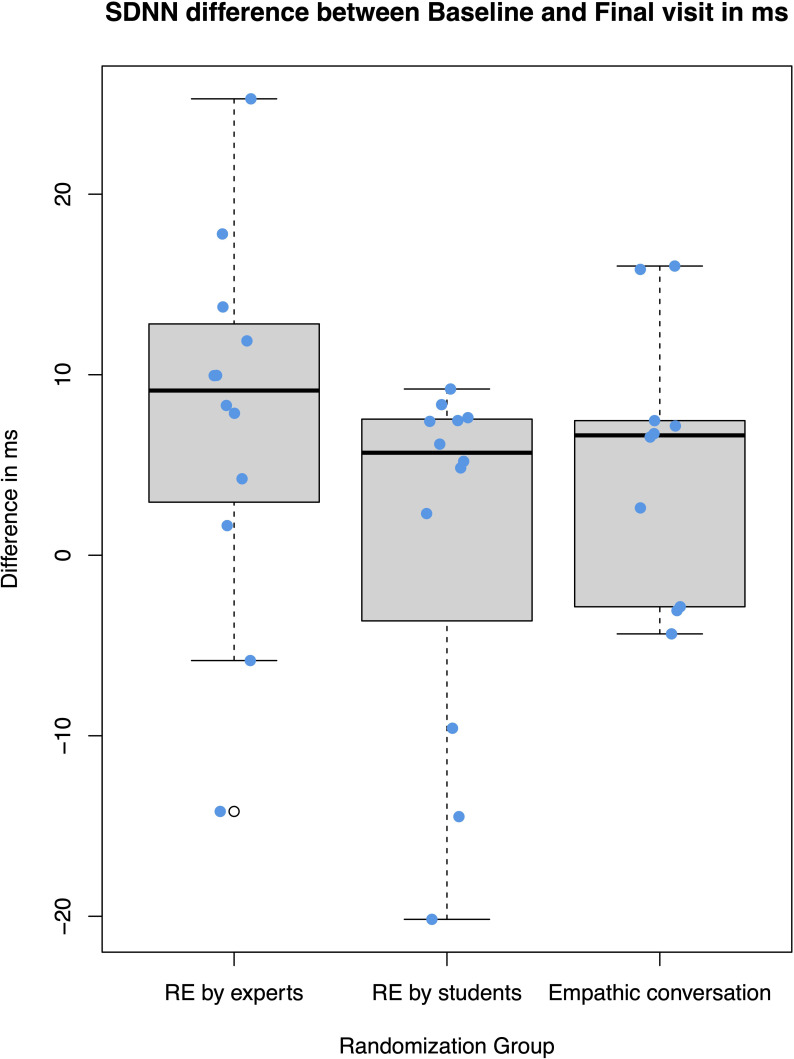
Boxplots showing SDNN difference between Baseline and Final visit in ms.

The primary endpoint, stress, was assessed by monitoring heart rate variability overnight from day 1 to day 2 and day 5 to day 6. The analysis was performed on an intention-to-treat basis using the full analysis set. However, many electrocardiogram recordings were affected by artifacts, resulting in data availability for 34 (50%) patients; comprising 12 from the expert RE group, 12 from the student RE group, and 10 from the empathic conversation group. Reasons for the artefacts were high skin resistance at the location of the electrodes in several patients and excessive movement in some patients. The mean SDNN of the final measurement was 46.4 ms (SD 20.0 ms) in the RE expert group, 36.6 (19.8) in the student RE group, and 42.1 (13.7) in the empathic conversation group. The Kruskal-Wallis-Test showed no statistically significant differences between the groups. The median change SDNN between the two measurements was an increase of 9.12 ms (IQR 3.59-12.3 ms) in the expert RE group, 5.68 ms (-0.66-7.5 ms) in the student RE group, 6.64 ms (-1.49-7.38 ms) in the empathic conversation group (see **Figure 2**); without statistical significant difference between the groups (Kruskal-Wallis: p= 0.21). For missing data of the HRV variables, we performed multiple imputation by chained equations in R; however, there were still no statistically significant effects between groups (for details, see the **Supplementary Material**).

#### 3.2.2 Per protocol analysis

Data of 26 patients were included in the per protocol analysis: 11 patients from the expert RE group, 8 from the student RE group, and 7 from the empathic conversation group. The median and IQR of the SDNN increase between the two measurements was 8.30 ms (2.94-10.9 ms) in the expert RE group, 5.68 ms (-0.664-7.5 ms) in the student RE group and 6.55 ms (-0.116-7.1 ms) in the empathic conversation group (Kruskal-Wallis: p=0.34).

#### 3.2.3 Secondary outcomes

**Figure 3:**
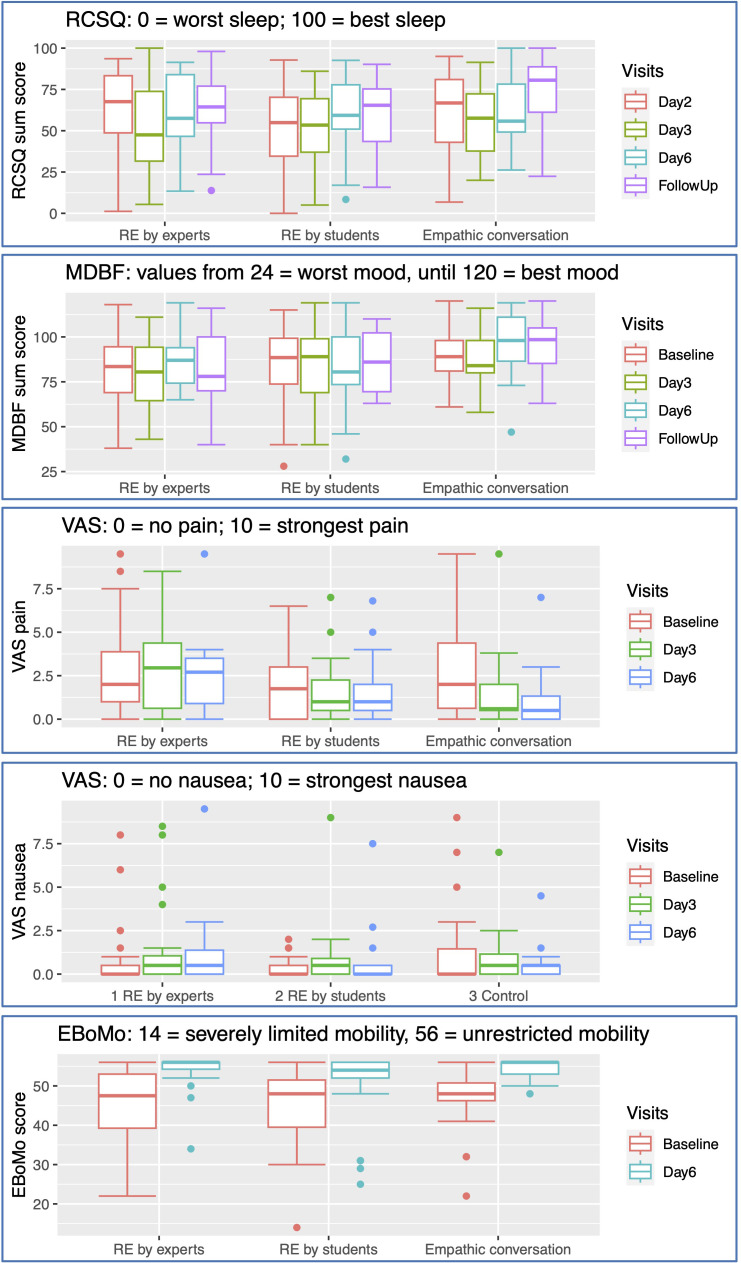
Secondary Outcomes: RCSQ, Richard-Campbells-Sleep-Questionnaire; MDBF, Mehrdimensionaler Befindlichkeitsfragebogen (multidimensional mood questionnaire); VAS, visual analogue scale; EBoMo, Evlauationsbogen Mobilität (evaluation sheet mobility).

Sleep quality was assessed using the Richards-Campbell-Sleep-Questionnaire (RCSQ). Across all groups, a decrease of sleep quality from night 1 to night 2 was observed (see **Figure 3**). The median difference of the RCSQ between the 2^nd^ and the 5^th^ night was 7.1 (IQR -9.40-28.3) in the RE expert group, 12.0 (-7.6-28.8) in the RE student group and 3.5 (-22.5-15.1) in the empathic conversation group; (Kruskal-Wallis: p=0.65).

MDBF at Baseline, day 3, day 6 and follow up after 30 days. The median improvement of mood from day 1 to day 6 was 3.5 (IQR -9.0-21.8) in the RE expert group, 4.5 (-7.5-8.0) in the RE student group: and 11.5 (0.5-17.8) in the empathic conversation group; (Kruskal-Wallis: p=0.31).

Pain intensity was generally rated low; median difference in pain scores between day 1 and day 6 were 0 in the RE expert group (IQR -2-1), -0.25 in the RE student group (-1.75-1.50) and -0.75 in the empathic conversation group (-4-0); (Kruskal-Wallis: p= 0.28).

Nausea was generally rated low; the median difference in nausea scores between day 1 and day 6 was 0.0 (IQR -0.5-0.5) in the RE expert group, 0.15 (0-0.925) in the RE student group and 0.0 (-0.500-0.125); (Kruskal-Wallis: p= 0.15).

Mobility was generally rated high; the median difference in mobility scores between day 1 and day 6 was 6.5 (IQR 3-16.3) in the RE expert group, 6. 0 (3.75-10.3) in the RE student group and 8.0 (2.75-9.25) in the empathic conversation group; (Kruskal-Wallis: p= 0.79).

The measurements of the secondary outcomes at the different visits are detailed in **Figure 3**.

Regarding pain medication, need of opioids at the final visit was not equally distributed between the groups. However; the high proportion of missing data renders this result inconclusive (for details see the figure on pain medication in the **Supplementary Material**). The overall use of sedatives was low in all groups (see **Supplementary Material**).

### 3.3 Complications

The study team assessed potential complications associated with the intervention at each patient encounter. No complications associated with the intervention were documented during the entire study period.

## 4 Discussion

The results of this study indicate that RE was safe and a significant superiority of RE over empathic conversations regarding the primary objective could not be found when compared with empathic conversation. Regarding the primary objective, a relevant part of the data were missing, due to technical issues of the HRV recording. Furthermore, the variability of the HRV results was higher than expected. In addition, groups were imbalanced regarding gender and tumor type, patients received effective treatments for pain, sleep, and nausea, which may have influenced the outcomes.

With regard to the HRV data, the technical difficulties encountered in the measurement of HRV resulted in the inability to analyze 50% of the data. Nevertheless, the extent of data loss was relatively evenly distributed across the groups. HRV can be calculated from short time periods of several minutes to record changes after an intervention, as Seifert et al. demonstrated in their trial on rhythmical massage in healthy young women (38). In our trial, we decided to calculate HRV from complete long-term measurements overnight to assess overall stress level rather than sudden changes. However, these long-term measurements are more susceptible to other stress triggers, which can vary in the clinical setting and throughout the course of a cancer disease. We did not ask about stress in another questionnaire, as we wanted to collect physiological parameters on this issue that would not be influenced by biases in the use of questionnaires.

Empathic conversation was chosen as a control intervention to differentiate between the mere effect of empathic caring time and the specific effect of RE. However, empathic conversations can have a strong effect on patients as well. Previous studies on empathic conversation have demonstrated changes in immunologic blood cells and a reduction in the duration of a common cold (39). Empirical evidence also exists demonstrating the impact of empathic conversation on stress, anxiety, and outcomes in chronic disease and cancer (40–45).

The Sars-CoV-2 pandemic introduced a number of significant alterations to the surrounding conditions of our trial. For instance, regular emphatic conversations were also conducted during the period of contact restrictions, where regular conversations with friends and family members were dramatically reduced. Consequently, the empathic conversations may have exerted a more profound influence than they would have done in the absence of the restrictions on regular visits.

The secondary endpoints were analyzed just for exploratory reasons. Also here, the sample displayed higher variability than expected and the groups were partially imbalanced. To investigate an effect, a larger sample size would be needed. For example, the tumor locations were distributed differently in the groups. Regarding the measurement of sleep quality, the first night at the normal ward unexpectedly was better than the following nights. However, this is a well-known phenomenon in sleep research, that a sleep dept can be developed in nights with disturbed sleep and a subsequent night with less disturbance – such as those at the normal ward after nights at the ICU – tend to be rated better than the following nights when no sleep debt is present (46). Rating of sleep in the second night at the normal ward was comparable to results from intensive care units and rating of the sleep at home in the follow up was comparable to sleeping at home from other populations (47). Mood was comparable to other studies of the perioperative mood change – although a definite trend within the postoperative period could not be seen in our trial (48). Patients’ pain, nausea and vomiting were closely monitored by the surgical ward and treated with medication. Therefore, it was difficult to discern any additional influence of the study interventions. We did not evaluate non-oncological comorbidities or factors that could potentially restrict the patients’ physical status. Given the small sample size, these unassessed factors may have further increased the heterogeneity of patient symptoms and outcomes.

The efficacy of massage interventions in the postoperative period has been the subject of various studies, which have yielded disparate results. Two studies with 58 and 113 patients conducted by the Mayo Clinic revealed that massage with additional resting time had a significantly positive impact on pain, anxiety, tension, and satisfaction in patients who had undergone cardiovascular surgery in comparison with resting time alone. This led to the incorporation of massage into the postoperative routine of the clinic (49). In contrast, a study conducted by the Cleveland Clinic found that postoperative massage had no effect on mood, pain, anxiety, or physiological measurements, with the exception of a reduction in postoperative blood pressure in patients undergoing cardiovascular surgery when compared to usual care alone (50). The discrepancy in these results can be attributed to a number of factors, such as individual tailoring of the massage treatment to the individual patient, type of massage and openness for touch and massage in general. Although participation in our trial was entirely voluntary, we did not inquire as to whether the patients would like to be massaged, whether they had previously had positive or negative experiences with massage, or whether they had any concerns about being touched by another person. To avoid amplifying expectation effects, we refrained from asking the participants whether they found the intervention pleasant. However, recent research on therapeutic touch indicates that the patient’s attitude toward the type of touch can be a determining factor in whether the therapy is perceived as calming or stressful (51, 52).

## 5 Conclusion

For future studies in RE, it is recommended that more robust and less error-prone measurement methods be employed. At the outset of the intervention, the post-operative stress had presumably already partially subsided, resulting in an effect size that was smaller than anticipated. The heterogeneity was considerable, and the number of test subjects should have been larger. Some symptoms were alleviated with medication, leaving little room for additional relief. Future studies should investigate areas that are insufficiently relieved by medication or at the time of greatest intensity. Previous experience with massage should be sought and included in the evaluation.

## Data Availability

The datasets presented in this article are not readily available because Data sharing was not included in the ethical review of the trial. Requests to access the datasets should be directed to Paul Werthmann, paul.werthmann@uniklinik-freiburg.de.
