# Supplementary material for: Efficacy and safety of massage for postoperative stress in colorectal cancer patients: a randomized, controlled, three-arm trial
Source: Front Oncol. 2025 Feb 5;15:1439420. doi: 10.3389/fonc.2025.1439420 (PMC11840018; doi:10.3389/fonc.2025.1439420)
Supplement: Supplementary file 1 [file DataSheet1.pdf]

Supplementary Material of the Article

"Efficacy and safety of massage for postoperative stress in colorectal cancer patients: a randomized, controlled, three-arm trial"

by Paul G. Werthmann, Dirk Cysarz, Melanie Jungbluth, Ann-Kathrin Lederer, Gergana Nenova, Roman Huber, Monique van Dijk, Gunver S. Kienle

Description of the intervention (rhythmic embrocation)

1. RE of the back in the morning:

The patient is sitting at the bedside and leaning with his arms on a nightstand or over a pillow; safe sitting has to be achieved in this position as an obligatory prerequisite for this treatment.

During this treatment, only one hand is touching the patient at one time. The treatment can be described as two different movements: in the first movement the hand is moved in circles over one side of the back with the circles going down; the circles are clockwise at the left side of the back and anti-clockwise at the right side of the back; there is a slightly stronger touch when going down above the M. erector spinae to the middle of back (the spine is not touched within these movements); the second movement is a line on one side of the back and then the other going down. Every movement is repeated about 3 times on every side of the back.

2. RE of the feet in the evening:

The patient is lying in his bed with a knee roll under his knees (or something similar like a rolled additional bedcover).

During this treatment the two hands of the nurse are touching one foot of the patient, either using both hands for the massage or one hand holding the foot and the other performing massage movements. The treatment can be described in 5 steps, though step 4 is a repetition of step 1. All steps are repeated 3-4 times and then the other foot is treated equally:

I. One hand is put into the arch of the foot, the other hand on the bridge. The hand on the arch induces a slight compression when moved towards the toes while the hand on the bridge follows with a lighter touch in the same direction – together gently wrapping the foot.

II. One hand lifts the foot slightly while supporting it in the area of the ankle. The other hand gives a slight impulse around the heel.

III. Both hands move in circles around the ankle and then over the bridge down to the toes.

IV. Repetition of I.

V. The thenar of the massage therapist touches the ball of the foot of the patient and moves with a slight impulse downwards along the arch to the heel. The other hand supports the bridge.

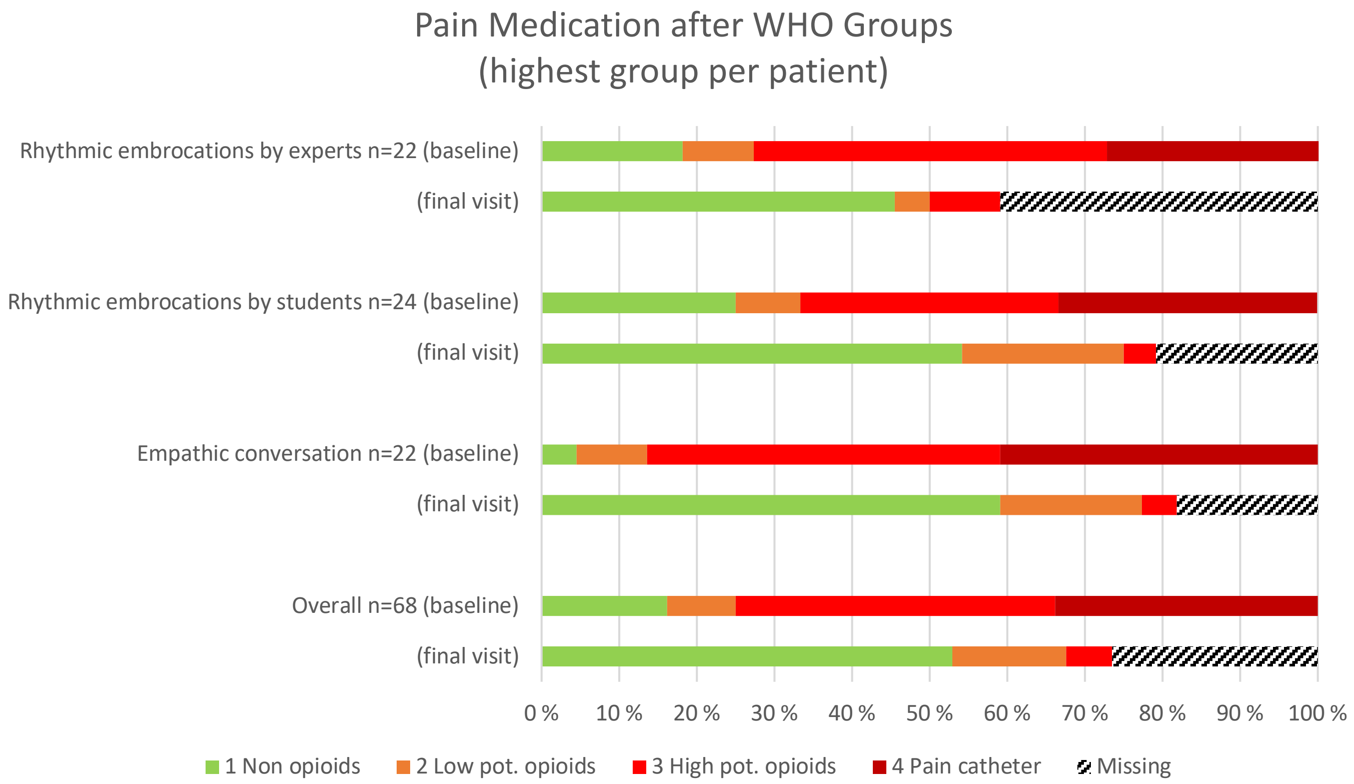
Pain Medication after WHO Groups
(highest group per patient)
Rhythmic embrocations by experts n=22 (baseline)
(final visit)
Rhythmic embrocations by students n=24 (baseline)
(final visit)
Empathic conversation n=22 (baseline)
(final visit)
Overall n=68 (baseline)
(final visit)
0 %
10 %
20 %
30 %
40 %
50 %
60 %
70 %
80 %
90 %
100 %
1 Non opioids
2 Low pot. opioids
3 High pot. opioids
4 Pain catheter
Missing

Sedatives per group at baseline and final visit

	Rhythmic embrocations by experts (n=22)	Rhythmic embrocations by students (n=24)	Empathic conversation (n=22)	Overall (n=68)
Sedatives at baseline	4 (18.2%)	3 (12.5%)	3 (13.6%)	10 (14.7%)
Sedatives at final Visit	1 (4.5%)	2 (8.3%)	0 (0%)	3 (4.4%)
Missings final visit	9 (40.9%)	5 (20.8%)	3 (13.6%)	17 (25.0%)

Heart rate variability data (full data set)

	RE by experts (N=22)	RE by students (N=24)	Empathic conversation (N=22)	Overall (N=68)
**SDNN Baseline**				
Median [IQR]	34.5 [23.9-55.6]	31.5 [18.1-40.4]	27.9 [22.8-51.6]	32.4 [21.9-51.6]
Missing	8 (36.4%)	10 (41.7%)	7 (31.8%)	25 (36.8%)
**SDNN final visit**				
Median [IQR]	44.1 [32.6-57.9]	29.6 [24.7-40.1]	39.1 [32.0-52.7]	38.9 [27.9-49.4]
Missing	8 (36.4%)	11 (45.8%)	10 (45.5%)	29 (42.6%)
**SDNN change**				
Median [IQR]	9.12 [3.59-12.3]	5.68 [-0.664-7.50]	6.64 [-1.49-7.38]	6.95 [1.81-8.99]
Missing	10 (45.5%)	12 (50.0%)	12 (54.5%)	34 (50.0%)
**RMSSD at Baseline**				
Median [IQR]	18.3 [15.0-24.1]	13.2 [8.12-25.4]	19.3 [14.1-20.4]	18.5 [11.9-23.5]
Missing	8 (36.4%)	10 (41.7%)	7 (31.8%)	25 (36.8%)
**RMSSD at final visit**				
Median [IQR]	26.7 [20.7-32.2]	18.6 [12.2-19.8]	18.6 [15.7-21.7]	20.4 [13.2-23.2]
Missing	8 (36.4%)	11 (45.8%)	10 (45.5%)	29 (42.6%)
**RMSSD change**				
Median [IQR]	7.07 [4.67-11.1]	1.34 [-5.19-3.60]	1.94 [-1.17-7.00]	3.06 [-1.66-8.44]
Missing	10 (45.5%)	12 (50.0%)	12 (54.5%)	34 (50.0%)
**pNN50 at Baseline**				
Median [IQR]	1.82 [0.733-5.38]	0.400 [0.249-6.56]	1.58 [0.387-3.39]	1.51 [0.375-5.33]
Missing	8 (36.4%)	10 (41.7%)	7 (31.8%)	25 (36.8%)
**pNN50 at final visit**				
Median [IQR]	7.24 [3.26-11.9]	2.97 [0.556-3.90]	1.94 [1.13-4.04]	3.22 [0.999-4.81]
Missing	8 (36.4%)	11 (45.8%)	10 (45.5%)	29 (42.6%)
**pNN50 change**				
Median [IQR]	3.90 [1.94-6.69]	0.200 [-3.57-0.710]	1.17 [0.161-3.03]	1.19 [-0.00592-3.53]
Missing	10 (45.5%)	12 (50.0%)	12 (54.5%)	34 (50.0%)
**pNN20 at Baseline**				
Median [IQR]	22.6 [16.7-38.8]	13.4 [3.66-31.5]	25.0 [14.2-31.1]	24.2 [8.94-33.1]
Missing	8 (36.4%)	10 (41.7%)	7 (31.8%)	25 (36.8%)
**pNN20 at final visit**				

	RE by experts (N=22)	RE by students (N=24)	Empathic conversation (N=22)	Overall (N=68)
Median [IQR]	38.3 [28.4-51.7]	12.4 [8.22-20.3]	21.4 [15.9-30.5]	22.9 [10.5-36.8]
Missing	8 (36.4%)	11 (45.8%)	10 (45.5%)	29 (42.6%)
**pNN20 change**				
Median [IQR]	14.5 [-0.0481-23.0]	1.46 [-10.1-5.08]	2.81 [-4.99-13.4]	3.23 [-4.99-15.6]
Missing	10 (45.5%)	12 (50.0%)	12 (54.5%)	34 (50.0%)
**VLF at Baseline**				
Median [IQR]	886 [438-2570]	823 [315-1410]	632 [311-2390]	844 [335-2360]
Missing	8 (36.4%)	10 (41.7%)	7 (31.8%)	25 (36.8%)
**VLF at final visit**				
Median [IQR]	1420 [882-2930]	727 [545-1670]	1510 [772-2540]	1090 [657-2350]
Missing	8 (36.4%)	11 (45.8%)	10 (45.5%)	29 (42.6%)
**VLF change**				
Median [IQR]	528 [33.1-1270]	148 [-27.2-299]	448 [308-667]	346 [49.0-669]
Missing	10 (45.5%)	12 (50.0%)	12 (54.5%)	34 (50.0%)
**LF at Baseline**				
Median [IQR]	275 [111-797]	134 [66.3-298]	191 [105-451]	191 [82.0-491]
Missing	8 (36.4%)	10 (41.7%)	7 (31.8%)	25 (36.8%)
**LF at final visit**				
Median [IQR]	347 [163-623]	231 [101-350]	257 [136-532]	284 [128-527]
Missing	8 (36.4%)	11 (45.8%)	10 (45.5%)	29 (42.6%)
**LF change**				
Median [IQR]	86.2 [-10.2-140]	35.3 [-48.1-64.7]	61.1 [-12.4-185]	53.0 [-22.8-119]
Missing	10 (45.5%)	12 (50.0%)	12 (54.5%)	34 (50.0%)
**HF at Baseline**				
Median [IQR]	144 [93.4-280]	87.7 [23.9-255]	90.7 [50.8-182]	109 [53.0-232]
Missing	8 (36.4%)	10 (41.7%)	7 (31.8%)	25 (36.8%)
**HF at final visit**				
Median [IQR]	266 [110-375]	81.0 [51.4-135]	128 [63.8-182]	135 [58.7-211]
Missing	8 (36.4%)	11 (45.8%)	10 (45.5%)	29 (42.6%)
**HF change**				
Median [IQR]	101 [25.5-140]	-4.73 [-104-19.1]	20.6 [-5.24-120]	21.3 [-19.6-102]
Missing	10 (45.5%)	12 (50.0%)	12 (54.5%)	34 (50.0%)
**LF/HF at Baseline**				
Median [IQR]	0.491 [0.164-1.06]	0.745 [-0.00979-1.12]	0.912 [0.471-1.11]	0.770 [0.0868-1.13]
Missing	8 (36.4%)	10 (41.7%)	7 (31.8%)	25 (36.8%)

	RE by experts (N=22)	RE by students (N=24)	Empathic conversation (N=22)	Overall (N=68)
**LF/HF at final visit**				
Median [IQR]	0.339 [-0.186-1.15]	1.11 [0.640-1.22]	1.01 [0.719-1.40]	0.843 [0.327-1.22]
Missing	8 (36.4%)	11 (45.8%)	10 (45.5%)	29 (42.6%)
**LF/HF change**				
Median [IQR]	-0.0626 [-0.525-0.0969]	0.113 [-0.0773-0.369]	0.199 [-0.482-0.378]	0.0118 [-0.347-0.379]
Missing	10 (45.5%)	12 (50.0%)	12 (54.5%)	34 (50.0%)

Heart rate variability data (per protocol population)

	RE by experts (N=11)	RE by students (N=8)	Empathic conversation (N=7)	Overall (N=26)
**SDNN Baseline**				
Median [IQR]	33.7 [22.8-53.2]	31.5 [19.7-42.8]	26.5 [19.7-39.9]	30.0 [21.5-50.0]
**SDNN final visit**				
Median [IQR]	47.2 [32.9-58.2]	39.4 [24.3-40.6]	33.0 [26.8-44.3]	36.4 [29.0-49.4]
**SDNN change**				
Median [IQR]	8.30 [2.94-10.9]	5.68 [-0.664-7.50]	6.55 [-0.116-7.10]	6.64 [1.81-8.33]
**RMSSD at Baseline**				
Median [IQR]	16.2 [15.0-23.9]	16.2 [9.02-27.5]	15.0 [11.7-20.0]	15.7 [12.6-24.1]
**RMSSD at final visit**				
Median [IQR]	30.2 [21.7-32.0]	19.5 [12.5-20.6]	20.4 [14.6-21.4]	20.5 [16.7-29.9]
**RMSSD change**				
Median [IQR]	5.86 [3.90-9.80]	-0.186 [-5.19-3.60]	1.50 [-0.905-7.51]	3.91 [-2.08-8.44]
**pNN50 at Baseline**				
Median [IQR]	1.51 [0.510-3.95]	1.42 [0.339-7.78]	0.980 [0.348-3.65]	1.42 [0.390-5.03]
**pNN50 at final visit**				
Median [IQR]	9.48 [3.48-11.9]	2.98 [0.963-3.66]	2.18 [1.21-4.60]	3.63 [1.43-9.49]
**pNN50 change**				
Median [IQR]	3.40 [1.92-5.64]	0.159 [-3.57-0.710]	0.936 [0.114-2.95]	1.25 [-0.173-4.13]
**pNN20 at Baseline**				
Median [IQR]	20.4 [16.8-38.4]	19.7 [3.33-34.0]	17.2 [8.66-26.3]	20.0 [11.2-36.2]
**pNN20 at final visit**				
Median [IQR]	39.6 [30.8-51.4]	15.5 [9.98-22.0]	19.9 [14.4-30.1]	27.2 [13.9-45.7]
**pNN20 change**				
Median [IQR]	12.0 [-0.574-21.1]	-2.52 [-10.1-5.08]	2.77 [-3.18-11.5]	3.62 [-4.99-16.2]
**VLF at Baseline**				
Median [IQR]	844 [409-2530]	971 [429-1650]	414 [311-1630]	637 [327-2180]
**VLF at final visit**				
Median [IQR]	1750 [889-2770]	1090 [618-1690]	772 [666-2050]	983 [696-2420]
**VLF change**				
Median [IQR]	402 [-6.58-935]	178 [-0.365-317]	419 [325-561]	363 [73.8-652]
**LF at Baseline**				

	RE by experts (N=11)	RE by students (N=8)	Empathic conversation (N=7)	Overall (N=26)
Median [IQR]	259 [95.8-720]	122 [65.5-523]	160 [66.9-290]	183 [66.3-520]
**LF at final visit**				
Median [IQR]	307 [169-793]	297 [104-397]	137 [128-403]	274 [127-566]
**LF change**				
Median [IQR]	76.5 [-13.6-163]	19.7 [-165-62.9]	33.1 [-47.0-121]	38.5 [-26.5-119]
**HF at Baseline**				
Median [IQR]	111 [94.2-212]	125 [25.2-237]	65.1 [42.2-103]	98.5 [58.4-190]
**HF at final visit**				
Median [IQR]	308 [131-366]	114 [56.5-161]	121 [62.3-179]	155 [70.0-290]
**HF change**				
Median [IQR]	103 [14.8-143]	-21.5 [-104-19.1]	39.9 [-11.7-108]	30.9 [-33.7-106]
**LF/HF at Baseline**				
Median [IQR]	0.443 [-0.281-0.866]	0.745 [0.352-1.10]	0.912 [0.702-1.01]	0.667 [0.351-1.07]
**LF/HF at final visit**				
Median [IQR]	0.0274 [-0.222-1.14]	1.04 [0.616-1.20]	0.815 [0.553-1.30]	0.771 [0.0413-1.19]
**LF/HF change**				
Median [IQR]	-0.0463 [-0.531-0.206]	0.132 [-0.0773-0.369]	0.203 [-0.320-0.441]	-0.00429 [-0.347-0.417]

Missing data handling

To impute missing data within the heart rate variability (HRV) data, we imputed data with the method of multiple imputations by chained equations (MICE) using the statistical software R, version 4.4.2 (2024-10-31) with the package mice version 3.17.0.

For the procedure we checked if all data types of the variables were coded correctly and the default settings for imputation method were used. We conducted the MICE procedure with 10 iterations, building 40 data sets. We checked the outcome of MICE with Kernel density estimates for the marginal distributions of the observed data.

The study results regarding the main outcome SDNN after the MICE procedure was as follows:

The mean SDNN of the final measurement was 47.7 ms (SD 23.0 ms) in the expert RE group, 41.7 (24.1) in the student RE group, and 45.6 (21.8) in the empathic conversation group. The Kruskal-Wallis-Test showed no statistically significant differences between the groups. The median change SDNN between the two measurements was an increase of 7.86 ms (IQR -5.83-13.8 ms) in the expert RE group, -3.61 ms (-14.5-8.15 ms) in the student RE group, 1.29 ms (-6.36-15.8 ms) in the empathic conversation group; without statistical significant difference between the groups (Kruskal-Wallis: p= 0.42).
